# Chronic Kidney Disease and Pulse Wave Velocity: A Narrative Review

**DOI:** 10.1155/2019/9189362

**Published:** 2019-02-17

**Authors:** Nicole Lioufas, Carmel M. Hawley, James D. Cameron, Nigel D. Toussaint

**Affiliations:** ^1^Department of Nephrology, The Royal Melbourne Hospital, Parkville, Australia; ^2^Department of Medicine (RMH), University of Melbourne, Parkville, Australia; ^3^Department of Medicine, Western Health, St Albans, Australia; ^4^Department of Nephrology, Princess Alexandra Hospital, Woolloongabba, Australia; ^5^Faculty of Medicine, University of Queensland, Woolloongabba, Australia; ^6^Monash Cardiovascular Research Centre, Monash Health, Clayton, Australia; ^7^Monash University, Clayton, Australia

## Abstract

Chronic kidney disease (CKD) is associated with excess cardiovascular mortality, resulting from both traditional and nontraditional, CKD-specific, cardiovascular risk factors. Nontraditional risk factors include the entity Chronic Kidney Disease–Mineral and Bone Disorder (CKD-MBD) which is characterised by disorders of bone and mineral metabolism, including biochemical abnormalities of hyperphosphatemia and hyperparathyroidism, renal osteodystrophy, and vascular calcification. Increased arterial stiffness in the CKD population can be attributed amongst other influences to progression of vascular calcification, with significant resultant contribution to the cardiovascular disease burden. Pulse wave velocity (PWV) measured over the carotid-femoral arterial segments is the noninvasive gold-standard technique for measurement of aortic stiffness and has been suggested as a surrogate cardiovascular end-point. A PWV value of 10 m/s or greater has been recommended as a suitable cut-off for an increased risk of cardiovascular mortality. CKD is a risk factor for an excessive rate of increase in aortic stiffness, reflected by increases in PWV, and increased aortic PWV in CKD shows faster progression than for individuals with normal kidney function. Patients with varying stages of CKD, as well as those on dialysis or with a kidney transplant, have different biological milieu which influence aortic stiffness and associated changes in PWV. This review discusses the pathophysiology of arterial stiffness with CKD and outlines the literature on PWV across the spectrum of CKD, highlighting that determination of arterial stiffness using aortic PWV can be a useful diagnostic and prognostic tool for assessing cardiovascular disease in the CKD population.

## 1. Introduction

Chronic kidney disease (CKD) is increasingly recognised globally as a major public health problem. CKD is defined as abnormalities of kidney structure and/or function, present for at least 3 months, the prevalence of which is estimated to be between 8 and 16% worldwide [1]. Patients with CKD have substantially increased morbidity and mortality compared to individuals without CKD, with the increase in disease burden and outcome events largely attributed to cardiovascular disease [2]. Considerable cardiovascular risk in the CKD population results not only from traditional cardiovascular risk factors, but also from atypical, CKD-specific cardiovascular risk factors including abnormalities of mineral and bone metabolism [3, 4].

Cardiovascular disease is the leading cause of death in CKD and a predominant driver for this is increased conduit artery stiffness, in particular of the elastic thoracic aorta. Increased carotid-femoral aortic stiffness, as measured by segmental pulse wave velocity (PWV), is a strong, independent predictor of cardiovascular mortality in this population [5, 6]. Although no current therapies are available, aortic stiffness is a potentially modifiable cause of cardiovascular dysfunction and a useful biomarker in risk stratification for patients with CKD. Previous studies have suggested that therapeutic modification of arterial stiffness may ameliorate cardiovascular mortality [7].

Carotid-femoral PWV is utilised as the noninvasive gold standard measure of arterial stiffness, with assessment of changes in vascular stiffness having been advocated as a suitable surrogate cardiovascular end-point in clinical trials. Development of vascular calcification and arterial stiffness observed with progression of CKD results in a different age-related pattern of PWV change than in the disease-free normal population. In addition, PWV has been demonstrated to characterise the progression of arterial disease in a CKD population [8, 9]. This review briefly outlines the pathophysiology of increased arterial stiffness in patients with CKD and discusses the use of PWV to assess arterial stiffness, as well as PWV changes mediated by interventions in clinical trials, in this population.

## 2. Pathophysiology of Aortic Stiffness and Cardiovascular Disease

Functional aortic stiffness is a result of both normal nonlinear pressure dependent changes in aortic wall properties and (usually irreversible) intrinsic structural changes. Increased aortic stiffness and its relatively easily measured manifestation, inappropriately increased PWV, result in the pathophysiological consequences of an increase in the magnitude of the aortic pressure waveform as well as other changes in the morphology of this wave including more rapid decay of diastolic blood pressure magnitude resulting in increased pulse pressure measured either centrally or at the brachial artery. These alterations in pressure wave morphology are associated with increased pulsatility exposure in feeding arteries to low impedance vascular beds, such as the kidneys and brain, with organ parenchyma exposed to high levels of mean blood pressure and increased mechanical strain, as well as being atherogenic in coronary arteries and associated with a decreased ability to augment coronary blood flow in hyperemic states.

There remain controversy and uncertainty regarding the predominant cause of these deleterious central blood pressure changes with conflicting hypotheses put forward. The traditional model of central pressure augmentation due to increased pressure wave reflection and from a predominant distal reflection site has been challenged by a more contemporary suggestion. This proposes that increased central blood pressure is predominantly due to increased proximal aortic stiffness and in fact decreased impedance mismatch distally which is caused by increased pulse pressure due to increased central pressure propagating distally [10].

Whatever the predominant underlying mechanism for the effect of increased central blood pressure and other changes in the pressure waveform associated with increased aortic stiffness, the clinical sequelae include increased cardiovascular and cerebrovascular events and deleterious effects on kidney function. Any primary renal effects that increase blood pressure will exacerbate the “vicious cycle” relationship between operating blood pressure and intrinsic aortic wall stiffness. Increased blood pressure causes increased functional stiffness due to the nonlinear volume pressure relationship of the aortic wall. Similarly, CKD or degenerative processes that primarily increase aortic stiffness will increase blood pressure.

## 3. Arterial Stiffness and Ageing

Arteries demonstrate a gradual, age-related impairment in vascular function likely related to reduction in endothelium-derived nitric oxide bioavailability and increased production of vasoconstrictors [11, 12]. Increased exposure and impaired ability for defence mechanisms to resist oxidative stress and inflammation may contribute to age-related changes in vascular function. Arteries also undergo structural changes with age including gradual thickening of the arterial wall, changes in wall content (advanced glycation end-products and less elastin), and an increase in conduit artery diameter [13]. These changes in structure have important interactive effects on artery function, with increases in small and large arterial stiffness representing a characteristic change with older age.

Increased aortic stiffness with ageing, and in association with disease processes, is often the result of medial degeneration, i.e., the process of* arteriosclerosis*, in contrast to* atherosclerosis*. Atherosclerosis is the underlying pathology of cardiovascular disease and is characterised by abnormalities in lipid metabolism leading to lipid-filled macrophage deposition in the subendothelial layers of the arterial wall, which results in chronic inflammation [14, 15]. Atherosclerotic disease contributes to the majority of vascular pathology in the general population with traditional Framingham cardiovascular risk factors, and different nonmodifiable risk factors such as genetic predisposition and ethnicity are also increasingly being understood.

Arteriosclerosis is associated with direct structural changes including elastin fragmentation and medial calcification but is also affected by interaction with cellular and molecular changes in the overlying intimal layer, i.e., the inflammatory atherosclerotic process. Increases in arterial stiffness with arteriosclerosis, independent of mean arterial pressure, result in end-organ damage by imposing hemodynamic stress on vascular beds, particularly vascular beds of low impedance and high flow. Different disease entities, in addition to ageing, contribute to arteriosclerosis and increased arterial stiffness dependent upon risk cohorts.

## 4. Pathophysiology of Arterial Stiffness and CKD

**Figure 1:**
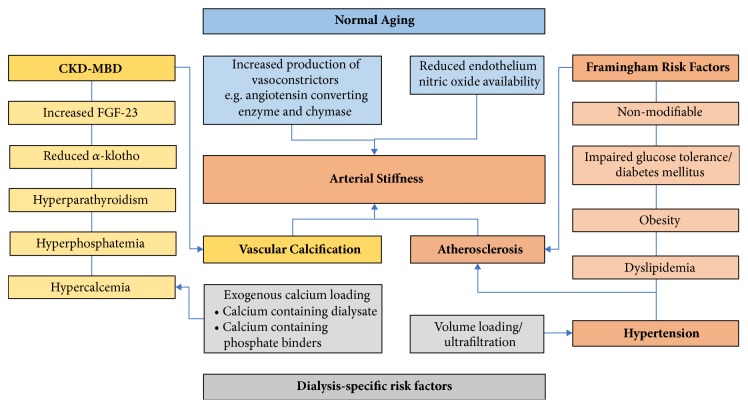
Pathophysiological factors contributing to arterial stiffness in patients with chronic kidney disease.** Abbreviations: **CKD-MBD, Chronic Kidney Disease–Mineral Bone Disorder; FGF-23, fibroblast growth factor 23.

Accelerated ageing is observed in patients with CKD and the mechanisms underlying arterial stiffness in CKD are extremely complex (Figure 1). One of the contributing factors is vascular calcification associated with Chronic Kidney Disease–Mineral and Bone Disorder (CKD-MBD). CKD-MBD is a complex entity comprising multiple mineralisation abnormalities, including hyperphosphatemia, hyper- and hypocalcemia, and hyperparathyroidism. As an active process, and in combination with a reduction in calcification inhibitors, deranged calcium and phosphate metabolism promotes vascular calcification in patients with CKD. Vascular calcification can either take place in the intima or in the media of the vessel wall. Calcification of the intima is a part of atherosclerosis, while medial calcification is the hallmark of arteriosclerosis. Both are prominent in CKD but arteriosclerosis primarily has an important role in the development of arterial stiffness in this population.

Elevated serum phosphate is a late manifestation of CKD and has been shown to accelerate mineral deposition in vessel walls leading to increased vascular calcification and arterial stiffness. *α*-Klotho and fibroblast growth factor 23 (FGF-23) are emerging factors in CKD-MBD and are thought to be involved in the pathogenesis of vascular calcification [16, 17]. There has been conflicting data regarding the relationship between PWV and FGF-23 however, despite the association of FGF-23 with progression in CKD and change in vascular calcification [18]. A recent observational study assessing PWV, aortic calcification, and bone mineral markers over a 12-month period in patients with advanced CKD reported a change in FGF-23 associated with changes in aortic calcification, although no change was seen in *α*-klotho [19]. There are changes in multiple other inhibitor and promotor proteins associated with the complex process of vascular calcification in patients with CKD, many of which are involved in normal bone formation. These include reduction in fetuin-A (a circulating inhibitor responsible for complexing with insoluble calcium-phosphate complexes), reduction in matrix Gla protein (another important inhibitor which can modify vascular calcification), and an increase in osteoprotegerin (which may inhibit vascular calcification through the RANK ligand pathway, although there is conflicting literature about its role) [20].

In patients with CKD, deleterious mechanisms are operative and lead to the increase in clinical manifestations of cardiovascular disease. The presence of CKD can be considered as both cause and effect of the increased aortic stiffness indicated by increased values of carotid-femoral PWV, which provides a potentially integrative pathophysiological mechanism linking CKD to increases in cardiovascular and cerebrovascular events.

## 5. Use of PWV to Determine Arterial Stiffness

PWV was initially described as a physiological concept by Crighton Bramwell in the early 1900s. He noted that the velocity of the pulse wave will have a proportional relationship to arterial wall tension and blood pressure [21]. With increasing technological advances in the later part of the century, a number of different techniques for determining vascular compliance were discovered. PWV can now be reliably measured by a combination of different tools which may include electrocardiography, blood pressure cuffs, and carotid tonometry. These methods estimate the time taken for the pulse wave to travel between two spaced arteries and subsequently determine a velocity measurement calculated from the elapsed time and distance between the two points. Most commonly, this is between the carotid and femoral arteries, although brachial ankle PWV has also been determined in studies [22]. Measuring PWV in vessels of closer proximity can be less accurate. Regardless of the site of measurement, correlations exist between different types of PWV measurement, including the use of magnetic resonance imaging (MRI) to determine PWV [23].

Ambulatory PWV determined over a 24-hour period has been validated against both invasive and noninvasive measurements of arterial stiffness [24, 25]. This 24-hour ambulatory method involves measurement of the brachial artery oscillometric blood pressure waves to estimate PWV and correlates to dynamic changes in blood pressure with activity throughout the day [26]. These ambulatory methods have been listed as a viable method for measurement of PWV in a statement by the American Heart Association on standardising vascular research in arterial stiffness [27].

## 6. PWV and Predictive Value in the Non-CKD Population

Reference values for PWV in a population with normal kidney function have been determined via a subgroup of the “Reference Values for Arterial Stiffness” Collaboration database, measuring PWV in 11,092 subjects [25]. This collaboration determined the difference in PWV according to cardiovascular risk factors, including smoking status, as well as for nonmodifiable risk factors such as age or gender. Of note, there is an observable increase in PWV of 1 m/s for every decade above midlife and PWV also increased by mean tertile of blood pressure [25]. Community-based cohorts such as AGES-Reykjavik and Framingham have also shown that PWV changes in the presence of other cardiometabolic risk factors, such as obesity, dysglycemia, hypertension, and hypercholesterolemia [28].

In the 2018 European Society of Cardiology Guidelines, a consensus PWV threshold of 10 m/s was reported as suggestive of increased cardiovascular risk and appropriate to be utilised to stratify intermediate risk patients (Grade 2b recommendation) [29]. This recommendation is based on the association between higher PWV with increased mortality and cardiovascular events. In a systemic meta-analysis of 17 original studies analysing cardiovascular events and mortality in relation to aortic PWV, the pooled relative risk for all-cause mortality was 1.15 for an increase in 1 m/s and 1.42 for an increase by one standard deviation (SD) [30]. A similar relative risk was also observed for cardiovascular mortality, with a predictive value independent of other traditional cardiac risk factors. Of note, this meta-analysis was all-inclusive, including patients in the general population at risk as well as higher risk subgroups, including patients with end-stage kidney disease (ESKD).

In a more recent meta-analysis of 16 studies (17,635 patients), Ben-Shlomo et al. assessed the predictive validity of aortic PWV for cardiovascular disease events beyond conventional cardiovascular risk factors and reported that PWV may enable better identification of high-risk populations who could benefit from more aggressive cardiovascular risk factor management [31]. After adjusting for conventional risk factors in this systematic review, PWV was a significant predictor of coronary heart disease (HR 1.23 [95% CI, 1.11 - 1.35]), stroke (HR 1.28 [95% CI, 1.16 - 1.42]), and cardiovascular events (HR 1.30 [95% CI, 1.18 - 1.43]).

Numerous other studies have evaluated the cardiovascular risk profile in patients with higher PWV. In a follow-up of the Rotterdam Study, aortic PWV, when separated into different tertiles, had a significant impact on cardiovascular mortality [32]. Coronary heart disease-free survival matched a model of logistic regression in the 3350 patients followed up over ten years in this study. Following from this premise, PWV has been shown to have a similar predictive value for other forms of large vessel atherosclerotic vascular disease, such cerebrovascular disease and peripheral vascular disease, although similar major cardiovascular end-points are utilised in studies [33]. PWV has also been shown to predict mortality and assist in prognostication for determining functional outcomes following an acute ischemic stroke [34], and an association is also seen with peripheral vascular disease patients who demonstrate increased mortality with higher tertiles of PWV. However, there are no studies to date which evaluate the predictive value of PWV on requirement of vascular intervention such as amputation [35].

More recent studies have evaluated the association between PWV and the development of cognitive impairment, independent of cardiovascular risk factors [36]. This relationship may be due to microvascular cerebral damage, associated with pressure effects on cerebral parenchyma in the context of increased central blood pressure secondary to increased aortic stiffness [37]. Increased PWV scores in longitudinal studies of those with cognitive impairment have been associated with poorer scores on verbal and cognitive testing, subclinical vascular brain injury, and in some studies, PWV has been an independent risk factor of cognitive decline [38, 39]. In one longitudinal cohort study, the odds of cognitive impairment increased by 40% in the context of higher tertiles of PWV [40].

## 7. PWV in the CKD Population

Patterns of PWV progression are different in the CKD population, with clinical manifestations of CKD often related to an accelerated ageing process and an increase in vascular calcification worsening with advancing CKD stages. Vascular calcification is reported to increase in both frequency and severity with deterioration in renal function, with an estimated 80-90% of patients having heavy calcific burden with CKD Stage 5D (on dialysis) [41–43]. Young patients, even those aged under 30 years, with advanced CKD or ESKD demonstrate a considerable prevalence of arterial calcification, which correlates with the significant cardiovascular burden in this population [42]. This accelerated aging process may also have a molecular basis, with reduced levels of soluble *α*-klotho in patients with CKD which are associated with increased arterial stiffness.

### 7.1. PWV in Nondialysis CKD Patients

CKD leads to an accelerated increase in PWV compared to the general population. Evaluation of carotid-femoral PWV in 150 patients with varying stages of CKD demonstrated early onset of elevated aortic stiffness and increased rate of progression over a year in CKD patients in contrast to those with normal kidney function, although in this study there was no significant change in PWV between different levels of kidney function [43]. In observational studies, change in progression of PWV by approximately 1 m/s each year is reported in patients with CKD [44], which represents an increased rate in progression of arterial stiffness in comparison to the normal ageing process [19]. Observational studies in the Rotterdam cohort also demonstrated an association between arterial stiffness and decline in kidney function, with an increased relative risk of CKD of 1.08 (95% CI, 1.03 - 1.14) for each SD higher PWV [45]. Increased PWV with greater vascular calcification is associated with higher mortality [46], perhaps due to the intimate relationship between blood pressure, vascular events, and the clinical entity of CKD known as renovascular disease.  Table 1 highlights a number of key observational trials in CKD cohorts which utilise PWV as an outcome.

A number of interventional trials have been conducted aiming to reduce the progression of arterial stiffness in patients with CKD and using PWV as a measure of arterial stiffness. The majority of studies aim to attenuate existing Framingham risk factors or proteinuria to improve arterial stiffness. Interventions have included eplerenone, enalapril, and candesartan in combination and atorvastatin (Table 2) [47–49]. Dual ACE/ARB inhibition demonstrated a small change in PWV over time, however counteracted by issues with progression of kidney disease and significant hyperkalemia leading to almost 15% of patients being withdrawn from this trial due to safety events. Other vascular interventional studies have only recruited small numbers of participants and are likely to be underpowered to demonstrate benefits on PWV.

One randomized controlled trial involving patients with CKD Stages 3b to 5 assessed the effect of vitamin D supplementation on arterial stiffness measured by PWV [60]. Patients were randomised to placebo, calcitriol, or calcifediol. In advanced CKD, there appeared to be a trend towards improvement over a 6-month period with vitamin D supplementation when adjusted for baseline, particularly in patients who were vitamin D deficient. However, there was also a significant reduction in parathyroid hormone, an expected normal physiological response to vitamin D supplementation, which may have affected the results. A smaller cohort evaluating the efficacy of two large pulsed doses of cholecalciferol (300,000 units) over 16 weeks did not demonstrate a significant change in PWV [58]. However, reductions were seen in biomarkers of endothelial function such as E-selectin, Intercellular Adhesion Molecule 1 (ICAM-1), and Vascular Cell Adhesion Molecule 1 (VCAM-1), which may suggest alterations to vascular remodelling. No significant change in FGF-23 was noted and limitations of this study included a short follow-up period and the study was underpowered to demonstrate significant alterations in PWV.

In an attempt to improve cardiovascular disease, the use of phosphate binders in nondialysis CKD patients has also been studied in randomised trials with assessment of changes in PWV as a surrogate end-point. These studies however, which have varied from 38 to 120 patients with CKD Stages 3 and 4 and involved varying phosphate binder treatment (lanthanum, sevelamer, and calcium-based binders) over 9- to 12-month periods, have not shown any change in PWV with this intervention [61, 62].

### 7.2. PWV in Dialysis Patients

Long-term cardiovascular outcomes of patients undertaking either peritoneal dialysis or conventional satellite hemodialysis are similar, but overall they are significantly worse compared to age- and gender-matched individuals in the general population [64]. Longer hours hemodialysis however has been associated with improved cardiovascular outcomes in observational trials, when compared to conventional hemodialysis, perhaps related to improved ultrafiltration rates and potentially better middle molecule clearance on dialysis [65].

Conflicting reports exist in observational studies regarding the impact of dialysis modality on arterial stiffness as measured by PWV [66, 67]. Studies present disparate results, likely in the setting of small patient numbers, although different dialysis modalities certainly all affect arterial stiffness. In a longitudinal study analysing differences in PWV over 12 months between patients on peritoneal dialysis and hemodialysis, increased progression of arterial stiffness was observed with hemodialysis [68]. The most significant difference between the cohorts was in residual renal function, with patients on hemodialysis predominantly anuric in contrast to more urine output in those on peritoneal dialysis (mean urine output recorded at 546.1 ± 365 mL daily). There is limited data otherwise regarding outcomes related to arterial stiffness in peritoneal dialysis. Reports of mortality outcomes for patients on satellite hemodialysis and peritoneal dialysis have been similar at a registry level and are likely due to the significant distribution of cardiovascular comorbidity in patients with ESKD rather than dialysis modality specific attenuation [69].

Hemodialysis causes significant changes in volume loading and blood pressure as a function of ultrafiltration. In observational studies, PWV is relatively stable during nondialysis days [70], although, at an individual level, PWV exhibits cyclical change with dialysis sessions including reduction after dialysis [71]. Higher PWV is particularly demonstrated after a three-day intradialytic break, in contrast to the two-day break [72]. One hypothesis is that excess volume amplifies aortic blood pressure and stiffness. Reduced mortality has been observed with quotidian home hemodialysis in contrast to satellite dialysis and daily dialysis dosing has been associated with a reduction in PWV, potentially by eliminating excess fluid load and electrolyte imbalances associated with dialysis breaks [71].

From a cardiac perspective, inappropriately increased PWV is associated with increased left ventricular mass in patients on hemodialysis, highlighting the mechanistic contribution of increased arterial stiffness to the development of left ventricular hypertrophy in this cohort [73]. Hemodiafiltration, postulated to reduce the stress of ultrafiltration and contribute to improved cardiac outcomes, has not been correlated with improvements in vascular stiffness in contrast to conventional hemodialysis [74]. A case-controlled study of 148 patients on hemodialysis and 141 on hemodiafiltration showed no change in brachial ankle PWV [75].

Vascular calcification in patients on dialysis is common, often extensive and progressive, and the causes are multifactorial. Contributing factors include the use of calcium-containing phosphate binders as well as higher calcium dialysate. Exogenous calcium loading has been associated with an increase in arterial stiffness in dialysis patients, independent of age, blood pressure, and dialysis vintage [76]. Higher calcium dialysate concentrations have also been associated with an increase in PWV, although patient numbers in these studies are small [77]. One small observational trial with a reduction of dialysate calcium from 1.75mmol/L to 1.5mmol/L in 20 patients on hemodialysis demonstrated improvement in PWV as well as serum calcification markers [78]. In fact, this dialysate calcium concentration is higher than most international practices and therefore it would be interesting to assess the effect of PWV with lower calcium concentrations, although this has not been studied. A small randomised unblinded study determined an interaction between time and dialysate calcium concentration in hemodialysis, again with increased PWV in those on higher dialysate calcium [79].

Several small studies have also assessed the impact of exercise programs in patients undertaking dialysis. Exercise is hypothesised to improve overall cardiovascular risk and potentially mitigates the impact of hypertension upon arterial stiffness. One cross-over study of 19 patients showed a trend towards improvement in PWV in patients who undertook interdialytic exercise, with subsequent deterioration in PWV after cessation of exercise [80]. Other trials in hemodialysis patients have failed to demonstrate any significant difference in PWV with exercise [81]. However, nonpharmacological interventions may potentially be beneficial to improve PWV in dialysis, as have been studied in other CKD cohorts with varying results [63].

### 7.3. PWV in Kidney Transplant Patients

Kidney transplantation improves cardiovascular prognosis, but its impact on arterial stiffness is still controversial. Kidney transplantation may potentially mitigate the development of further worsening of arterial stiffness [82], and donor age, living kidney donation, and mean blood pressure appear to be the main determinants of improvement in arterial stiffness after kidney transplantation. The predictive value of PWV for cardiovascular mortality has also been reported in kidney transplant patients, and there appears to be an attenuation of the usual increased PWV seen with advanced CKD [83]. In a prospective cohort of 66 kidney transplant patients, there was no significant change in aortic PWV in the first year posttransplantation, in contrast to the majority of studies demonstrating progression of arterial stiffness in patients with CKD over the same timeframe [82]. Several small studies suggest an improvement in carotid intima media thickness as well as PWV following kidney transplantation in relation to age-matched dialysis controls. PWV values in transplant recipients correlate with previous dialysis vintage and changes in PWV with any intervention, consistent with lack of reversal of structural changes, do not reduce to values seen in the general population [84].

## 8. Further Research

The prognostic significance and change in PWV in the CKD population need further investigation. The influence of dialysis modality upon PWV requires further evaluation, including the assessment of progression of arterial stiffness with different dialysis modalities. The contribution of residual kidney function, arterial turbulence, and fluctuations in volume state to changes in PWV also warrant further investigation. It has been hypothesised that peritoneal dialysis causes less hemodynamic and cardiovascular stress than hemodialysis; however, differences in mortality and cardiovascular outcomes have not been shown. Changes in PWV measured in carefully conducted trials would be helpful to determine the contribution of arterial stiffness in patients with ESKD.

Additionally, improvement in arterial stiffness in kidney transplantation should be investigated further. Kidney transplantation is the gold standard therapy for ESKD and is associated with reduced mortality, in part by mitigating the rate of vascular calcification due to restored calcium phosphate homeostasis. However, kidney transplant recipients have multiple cardiometabolic risk factors which still contribute to development of cardiovascular disease. PWV could potentially be utilised as a study end-point to assess the effect of interventions in improving overall cardiovascular outcomes in the context of graft dysfunction and posttransplant diabetes in this cohort. Of interest would be to study the use of newer hypoglycaemic agents, such as SGLT-2 inhibitors, in posttransplant diabetes with measurement of PWV as a surrogate cardiovascular end-point, although these medications would be used with caution in kidney transplant recipients with impaired kidney function.

Recently, new oscillometric methodologies using simple brachial cuffs, such as Mobil-O-Graph, have been introduced in patients with CKD to measure parameters of 24-hour arterial stiffness including PWV. This enables study of the 24-hour variability of these parameters, which will hopefully lead to better cardiovascular risk stratification and improved cardiovascular outcomes of CKD patients [85].

In conclusion, PWV is increased in CKD compared to healthy age-matched populations. It provides a unique, noninvasive clinical tool for determining arterial stiffness and hypertensive end-organ damage. Multiple studies have reported the predictive value of heightened PWV in the development of any major vascular disease. In the CKD cohort, studies utilising PWV have demonstrated greater progression of arterial stiffness, in contrast to the general population with normal kidney function, corresponding to an increase in cardiovascular mortality. Factors such as CKD stage, exogenous calcium load, dialysate calcium, volume status, and dialysis modality may all contribute to progression of PWV. Further interventions to reduce the burden of cardiovascular disease in the CKD population are essential and could be studied initially using PWV measurement as a surrogate marker.

## Figures and Tables

**Table 1:** Prospective observational studies assessing PWV in patients with CKD.

**Study**	**Population and follow-up**	**Outcome**
Zoungas *et al*, 2007 (ASFAST) [6]	315 CKD Stage 4–5, median 42 months follow-up	PWV predictive of composite end point of cardiovascular events; HR for every 1 m/s increase in PWV was 1.18 (95% CI, 1.12 - 1.25)
Chen *et al*, 2012 [50]	186 CKD Stage 3–5 patients, mean 22 months follow-up	PWV associated with increased rate of CKD progression
Tholen *et al*, 2013 [44]	70 CKD Stage 2–4, 12 months follow-up	Increase in PWV over 1 year by 1.1 m/s
Baumann *et al*, 2014 [51]	135 CKD Stage 2–4, 42 months follow-up	Aortic PWV ≥ 10 m/s an independent predictor of mortality; HR 5.1 (95% CI, 1.1 - 22.9)
Chandra *et al*, 2014 [52]	240 CKD Stage 3–5, up to 48 months follow-up	Low heart rate variability and high PWV associated with increase in first cardiovascular event; HR 1.19 (95%CI, 1.09 - 1.31)
Townsend *et al*, 2015 [9] (CRIC study)	2800 CKD, 7-10 years follow-up	Increase in PWV according to CKD Stage by up to 1 m/s per year; increasing PWV associated with cognitive impairment, mortality, heart failure admissions.
Cseprekal *et al*, 2016 [53]	94 CKD Stage 1–5 patients, median 52 months follow-up	PWV related to cardiovascular events; other associations included increasing calciprotein particles
Krishnasamy *et al*, 2017 [19]	42 CKD Stage 4 vs 40 healthy controls, 12 months follow-up	CKD associated with higher PWV (9.7[7.6-11.7] vs 8.1[7.2-9.7] m/s), with mean progression of PWV 1.3 m/s over 12 months
Kim *et al*, 2017 [54, 55] (KNOW-CKD)	2238 CKD Stage 1–5, over 10 years follow-up	Higher PWV with increasing CKD stage; also associated with increased aortic calcification and reduced bone mineral density

**Abbreviations: **CI, confidence interval; CKD, chronic kidney disease;HR, hazard ratio;PWV, pulse wave velocity.

ASFAST, Atherosclerosis and Folic Acid Supplementation Trial.

CRIC, Chronic Renal Insufficiency Cohort.

KNOW-CKD, KoreaN Cohort Study for Outcomes in patients With Chronic Kidney Disease.

**Table 2:** Interventional trials in the non-dialysis CKD population using PWV as a reported outcome.

**Study**	**Population and follow up**	**Intervention**	**Outcome**
**Lipid-lowering trials**			
Fassett *et al*, 2010 [47] (LORD)	37 CKD Stage 2–4, >3 years follow-up	Atorvastatin 10mg daily vs placebo	48% slower increase in PWV with atorvastatin (not significant)
Morita *et al*, 2014 [56]	37 CKD Stage 2–5, >24 weeks follow-up	Ezetimibe 10mg daily	Significant reduction in brachial ankle PWV (1,770.4±590.3 cm/s to 1,702.5± 519.9 cm/s), but no correlation with LDL levels
**Anti-hypertensive trials**			
Edwards *et al*, 2009 [57]	112 CKD Stage 2–3, 40 weeks follow up	Spironolactone 25mg daily vs placebo	Improvement in PWV -0.8 +/- 1.0m/s vs -0.1 +/- 0.9m/s
Frimodt-Møller *et al*, 2012 [48]	67 CKD Stage 3–5, 24 weeks follow-up	Monotherapy with ACEI or ARB, then randomised to dual therapy	Improved arterial stiffness in dual RAS blockade cohort (PWV -0.3m/s)
Boesby *et al*, 2013 [49] ALBLOCK-2	54 CKD Stage 3–4, >24 weeks follow-up	Open label trial of eplerenone vs placebo	No effect on PWV; reduction in pulse wave reflection with eplerenone
**CKD-MBD Modulation trials**			
Chitalia *et al*, 2014 [58]	26 CKD Stage 3–4, 18 weeks follow up	Vitamin D supplementation 300,000 units at baseline and 8 weeks	No significant effect on arterial stiffness; change in endothelial biomarkers with reductions in ICAM-1, VCAM-1
Kumar *et al,* 2017 [59]	120 CKD Stage 2–4, 16 weeks follow-up	Cholecalciferol 300,000 IU at baseline and 8 weeks vs placebo	Significant reduction in PWV with cholecalciferol (-1.24 m/s over 16 weeks)
Levin *et al,* 2017 [60]	87 CKD Stage 3b-4, 6 months follow-up	Vitamin D 5000 IU daily, calcitriol vs placebo	Non-significant change in PWV with fixed dose cholecalciferol
Seifert *et al*, 2013 [61]	38 CKD Stage 3, 12 months follow-up	Lanthanum carbonate vs placebo	Non-significant change in PWV
Chue *et al*, 2013 [62] (CRIB-PHOS)	120 non-diabetic CKD Stage 3, 40 weeks follow-up	Sevelamer carbonate vs placebo	No change observed in PWV
**Non-pharmacological trials**			
Van Craenenbroek *et al, *2015 [63]	48 CKD Stage 3–4, 3 months follow-up	Parallel group design, 3-month home based aerobic training program (4 daily training sessions of cycling) vs control	No significant change in PWV

**Abbreviations: **CKD, chronic kidney disease;ICAM-1, Intercellular Adhesion Molecule 1; LDL, low-density lipoprotein;PWV, pulse wave velocity; VCAM-1, Vascular Cell Adhesion Molecule 1.

ALBLOCK-2, Aldosterone Blockade in CKD.

LORD, Lipid Lowering and Onset of CKD trial.
